# Inhibition of GPX4 by *Toxoplasma gondii* Promotes Ferroptosis and Enhances Its Proliferation in Acute and Chronic Infection

**DOI:** 10.3390/cells14100756

**Published:** 2025-05-21

**Authors:** Yanlong Gu, Zhipeng Niu, Hui-Hong Lu, Si-Ang Li, Dong-Hui Zhou

**Affiliations:** Key Laboratory of Fujian-Taiwan Animal Pathogen Biology, College of Animal Sciences, Fujian Agriculture and Forestry University, Fuzhou 350002, China

**Keywords:** *Toxoplasma gondii*, ferroptosis, glutathione peroxidase 4 (GPX4), lipid peroxidation, reactive oxygen species (ROS)

## Abstract

*Toxoplasma gondii* (*T. gondii*) is an intracellular parasite that extensively infects warm-blooded animals, causing toxoplasmosis and posing a significant threat to global public health. In this study, we investigated the association between *T. gondii* infection and ferroptosis in host cells, as well as the regulatory role of glutathione peroxidase 4 (GPX4). Our findings revealed that mice infected with RH and PRU strains of *T. gondii* exhibited significantly elevated levels of reactive oxygen species and malondialdehyde in brain and liver tissues. Concurrently, the expression of GPX4, a critical negative regulator of ferroptosis, was downregulated, which correlated with the elevated parasite burden. In Vero cells, *T. gondii* infection similarly inhibited GPX4 expression, whereas GPX4 overexpression suppressed *T. gondii* proliferation. These results indicate that *T. gondii* infection can promote ferroptosis in host cells and that GPX4 plays a pivotal role in regulating infection and proliferation. This study provides novel insights into the pathogenic mechanisms of *T. gondii* and identifies GPX4 as a regulatory factor that constrains parasite proliferation, offering new approaches for toxoplasmosis prevention and control.

## 1. Introduction

*Toxoplasma gondii* is an obligate intracellular parasite belonging to the phylum Apicomplexa, capable of infecting nearly all warm-blooded animals, including humans, and causes zoonotic toxoplasmosis [1,2]. This parasite is globally prevalent [3]. In China, *T. gondii* infection is highly prevalent among farmed animals, particularly in pigs, sheep, and chickens. Surveys show that the seropositive rate of these animals is approximately 23.7% [4,5]. Among companion animals, such as cats, the infection rate in China shows a more severe situation compared to the global scale. The positive rate of *T. gondii* in domestic cats and stray cats is as high as 79.4% [4]. With an epidemiological survey indicating that approximately one-third of the global population is infected [3], there has been a consistent upward trend in the positive rate of human infection with *T. gondii* in China over recent decades, reaching a positivity rate of approximately 8.2% [5,6]. The increasing trend in the serum positivity rate highlights the pressing necessity to address the health risks associated with *T. gondii* infection among immunocompromised individuals and pregnant women. In immunocompetent individuals, *T. gondii* infection is typically asymptomatic, but it can be fatal for immunocompromised or immunodeficient individuals. Moreover, congenital transmission during pregnancy can lead to fetal deformities or death [7]. Beyond these clinical manifestations, *T. gondii* infection also disrupts host cellular and metabolic homeostasis. Among the various affected metabolic processes, the alteration in host iron homeostasis is especially noteworthy. Prior research has demonstrated that *T. gondii* infection indeed influences the host’s iron homeostasis [8]; however, the regulatory mechanism between this disruption and ferroptosis has yet to be elucidated.

Given the critical role of iron deficiency or excess on host health, the regulation of iron levels in organisms has emerged as a topic of considerable research value. To prevent the detrimental accumulation of iron ions within cells, the body must exert precise and stringent control over iron absorption, utilization, and storage [9]. Ferroptosis is an Erastin-induced, iron-dependent, and peroxidation-driven regulated form of cell death that was first described by Dixon et al. [10] in 2012. Ferroptosis is characterized by distinct mitochondrial ultrastructural changes, including atrophy, outer membrane rupture, and loss of cristae, as well as redox imbalance [11]. These alterations are driven by iron accumulation, excessive production of reactive oxygen species (ROS), and the depletion of glutathione (GSH) and glutathione peroxidase 4 (GPX4)-mediated antioxidant defenses, ultimately leading to lipid peroxidation-induced cell death [12,13,14,15].

The formation of ROS and subsequent lipid peroxidation, primarily induced by hydroxyl radicals (OH), constitute a critical pathway in ferroptosis [10,16,17]. This process is mitigated by the cellular antioxidant system. Specifically, within the context of oxidative stress, the System Xc-/GSH-GPX4 axis serves as a fundamental component of both enzymatic and non-enzymatic antioxidant defense mechanisms that counteract ferroptotic cell death [18,19]. GPX4 is a pivotal gene in the regulation of ferroptosis, specifically eliminating phospholipid peroxides such as cholesterol and polyunsaturated fatty acids (PUFAs) [20,21,22]. GSH acts as a co-factor for GPX4, assisting in the removal of lipid peroxides and exerting antioxidant effects. GPX4 catalyzes the reduction of GSH with free hydrogen peroxide or organic hydroperoxides to produce water or corresponding alcohols, thereby mitigating ROS production and reducing cellular damage caused by lipid peroxidation, thus protecting cells [23,24].

As an emerging form of regulated cell death, the discovery of ferroptosis has broadened the horizons for investigating parasitic diseases. Currently, functional studies on ferroptosis predominantly focus on cancers and metabolic disorders, with limited research dedicated to the functional implications of ferroptosis in parasitic infections [25,26]. In this study, we employed both in vivo and in vitro models to investigate whether infection with RH and PRU strains of *T. gondii* induces ferroptosis in host cells. Specifically, we measured the levels of ROS and malondialdehyde (MDA), which are key markers of ferroptosis, and observed that these levels were elevated following *T. gondii* infection. Additionally, the expression of GPX4, a critical negative regulator of ferroptosis, was downregulated, while the parasite burden increased. These findings suggest that *T. gondii* infection can induce ferroptosis in host cells. Furthermore, the overexpression of GPX4 was found to inhibit the proliferation of *T. gondii*. Our results elucidate the pathogenic mechanisms of *T. gondii* and highlight the pivotal role of GPX4 in regulating infection and proliferation. These insights will facilitate the development of more targeted strategies for controlling *T. gondii*.

## 2. Materials and Methods

### 2.1. Ethics Statement

The animal protocol used in this study was approved by the Regulation of College of Animal Sciences, Fujian Agriculture and Forestry University of Research Ethics Committee (Permit Number: PZCASFAFU22022). All mouse experiments were carried out according to the Regulations for the Administration of Affairs Concerning Experimental Animals in accordance with the State Council of the People’s Republic of China.

### 2.2. Laboratory Animals, Parasites

Specific pathogen-free (SPF) grade female C57BL/6 mice, aged 6 to 8 weeks, were procured from Spife Biotechnology Co., Ltd. (Beijing, China). *T. gondii* RH and PRU strains were maintained in our laboratory.

### 2.3. Cell Culture and Treatment

The Vero cells utilized in this experiment were stored at −80 °C in the laboratory. For resuscitation, the cells were rapidly thawed at 37 °C and subsequently cultured in Dulbecco’s Modified Eagle’s Medium (DMEM) (Thermo Fisher Scientific, Waltham, MA, USA) supplemented with 10% fetal bovine serum (FBS) (Thermo Fisher Scientific, Waltham, MA, USA) and 1% penicillin–streptomycin. Cells were cultured at 37 °C with 5% CO_2_.

### 2.4. Parasite Culture, Infection Model Construction, and Sample Collection

The cryopreserved RH strain was retrieved from liquid nitrogen storage and maintained through serial intraperitoneal passages in BALB/c mice [27]. The *T. gondii* PRU strain was maintained in BALB/c mice through the oral route. The brains of the infected mice were subsequently extracted and homogenized in saline for use in subsequent experiments [28].

For in vivo infection, RH tachyzoites were collected, filtered, purified, and quantified. The experiment comprised one control group and six experimental groups, each group containing three mice. Each mouse in the experimental groups was inoculated with 10^3^ tachyzoites. The mice were humanely euthanized at 6, 12, 24, 48, 72, and 96 h post-inoculation. For the establishment of the PRU strain infection model, the experiment included one control group and three experimental groups, each group consisting of nine mice. Mice in the experimental groups were intragastrically inoculated with 20 *T. gondii* PRU cysts. The mice were humanely euthanized at 7, 14, and 21 days post-infection. Liver and brain tissues were aseptically harvested and stored at −80 °C for subsequent analyses.

For in vitro infection, revived Vero cells were passaged to the third generation. Following this, the purified RH strain of *T. gondii* was diluted to a concentration of 10^3^ tachyzoites per 200 μL. The original medium in the six-well plates was aspirated, and the wells were washed three times with PBS before being replenished with culture medium containing 1% fetal bovine serum. Each well received 1 × 10^3^ *T. gondii* tachyzoites, which were gently mixed to ensure even distribution. After infection periods of 6, 12, 24, 48, and 72 h, samples were collected for subsequent experiments.

### 2.5. RNA Extraction and cDNA Synthesis

Total RNAs were extracted using the RNAsimple Total RNA Kit (TIANGEN BIOTECH, Beijing, China) according to the manufacturer’s instructions. Subsequently, the concentration of the extracted RNA was quantified using a NanoDrop 2000 spectrophotometer (Thermo Fisher Scientific, Waltham, MA, USA), and its integrity was assessed via 1.5% agarose gel electrophoresis. The cDNA template for RT-qPCR was synthesized using a PrimeScript^TM^ RT reagent Kit with gDNA Eraser (Takara, Dalian, China) and stored at −20 °C.

### 2.6. Plasmid Construction

The primer sequences were specifically designed based on the GPX4 gene sequence. Corresponding restriction sites (*Pst I* and *Xba I*, indicated by underlined letters) and protective nucleotides were incorporated into the primer sequences, which were then synthesized for the amplification of the target genes. The primer sequences utilized in this study are detailed in Table 1. The target fragment was purified using the TIANgel Midi Purification Kit (TIANGEN BIOTECH, Beijing, China) and subsequently cloned into the pMD^TM^19-T (Takara, Dalian, China) plasmid. The GPX4 plasmid and the pVAX1 eukaryotic expression vector (Invitrogen, Waltham, MA, USA) were subjected to double digestion at 37 °C for 2 h. T4 DNA ligase (Takara, Dalian, China) was utilized for ligation, and the resulting construct was transformed into *E. coli* DH5α-competent cells (Biomed Gene technology Co., Ltd., Beijing, China). Positive clones were selected and confirmed by sequencing. The correctly sequenced construct was designated as pVAX-GPX4 and stored for subsequent experiments.

### 2.7. Cell Transfection and Indirect Immunofluorescence (IIF)

The pVAX1 empty plasmid and the pVAX-GPX4 plasmid were extracted using the EndoFree Mini Plasmid Kit II (TIANGEN BIOTECH, Beijing, China). Subsequently, the plasmids were transfected into Vero cells following the protocol provided in the Beyotime Lipo8000^TM^ Transfection Reagent (Beyotime Biotechnology, Shanghai, China) for protein expression. After 24 h, the cells were fixed using 4% paraformaldehyde and subsequently incubated with GPX4 antibody (Abcam, Cambridge, UK) at 37 °C for 2 h. This was followed by incubation with goat anti-rabbit IgG antibodies conjugated to Fluor488 (Affinity Biosciences, Cincinnati, OH, USA) for 1 h at 37 °C as secondary antibodies. The samples were then mounted on slides and imaged using fluorescence microscopy for analysis.

### 2.8. Quantitative Real-Time Polymerase Chain Reaction (RT-qPCR)

RT-qPCR testing was conducted in strict adherence to the manufacturer’s protocol (Takara, Dalian, China). The Ct values of the internal reference gene β-actin (Table 1) were used to normalize the obtained sample data. Relative expression differences were calculated using the 2^−ΔΔCT^ method, and statistical significance was assessed using the Student’s *t*-test [29].

For the detection of parasite load, we utilized the RT-qPCR method. Initially, total RNA from *T. gondii* was extracted. Plasmids containing fragments of the B1 gene were used as standard templates and serially diluted to concentrations ranging from 10^8^ to 10^2^ copies/μL. RT-qPCR was conducted for each dilution. The amplification curves were validated for their validity and specificity, and a standard curve was subsequently established. The expression levels of *T. gondii* B1 gene were quantified at various time points using RT-qPCR. The expression levels were input into the standard curve derived from the *T. gondii* B1 RT-qPCR assay to obtain the final results [30].

### 2.9. Enzyme-Linked Immunosorbent Assay (ELISA)

The levels of ROS, MDA, GSH, and glutathione peroxidase (GPx) activity in mouse tissues were quantified using ELISA according to the protocol provided by Shanghai Enzyme-linked Biotechnology Co., Ltd. (Shanghai, China). Specifically, tissue homogenates were prepared from mouse samples, and the supernatant was collected following centrifugation. The sample was subsequently diluted by a factor of five and transferred to the microplate. Biotinylated antibodies were promptly added, and the mixture was incubated at 37 °C for 1 h. Subsequently, the wells were washed three times with a washing buffer, followed by the addition of streptavidin–HRP conjugate. The microplate was gently mixed and incubated at 37 °C for 30 min. Finally, the chromogenic substrate was added and allowed to develop for 10 min before the reaction was stopped with a stop solution. The optical density (OD) values were measured at 450 nm using a microplate reader (Thermo Fisher Scientific, Waltham, MA, USA).

### 2.10. Western Blotting (WB)

Cell and tissue samples were lysed in RIPA lysis buffer (Beyotime Biotechnology, Shanghai, China) containing Phenylmethylsulfonyl fluoride (PMSF). The supernatant was collected by centrifugation at 10,000× *g* for 10 min at 4 °C. The protein sample was mixed with the 1 × loading buffer (Takara, Dalian, China) and boiled at 95 °C for 10 min to ensure complete denaturation. Subsequently, the SDS-PAGE analysis was carried out. Following electrophoresis, the proteins were transferred onto a polyvinylidene fluoride (PVDF) (Beyotime Biotechnology, Shanghai, China) membrane using the wet transfer method. Before transfer, the PVDF membrane was pre-activated in methanol. Post-transfer, the membrane was blocked with 5% skim milk by shaking horizontally at room temperature (60 rpm) for 1.5 h. After blocking, the membrane was washed with TBST. It was then incubated with the primary antibody at 4 °C overnight. Following this, the membrane was incubated with the pre-diluted secondary antibody at room temperature for 1 h while shaking. Finally, chemiluminescence detection was employed for image acquisition.

### 2.11. Statistical Analysis

All statistical analyses were conducted using GraphPad Prism version 5.0. For comparisons between two groups, an unpaired two-tailed *t*-test was employed. For comparisons among multiple groups, one-way analysis of variance (ANOVA) was utilized. Results were presented as mean ± standard deviation (SD). The following *p*-value thresholds were applied for all tests: * *p* < 0.05, ** *p* < 0.01, *** *p* < 0.001, and **** *p* < 0.0001.

## 3. Results

### 3.1. T. gondii Infection Elevated the Levels of ROS and MDA in Mouse Tissues

**Figure 1:**
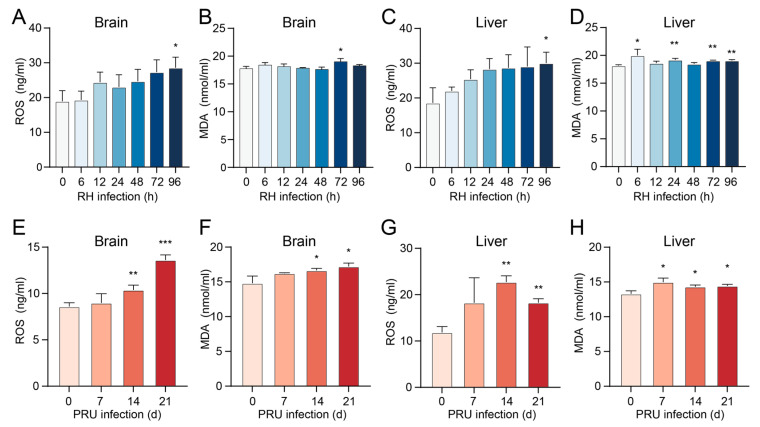
Expression levels of ROS and MDA in the brain and liver tissues of mice infected with *T. gondii*. (**A**,**B**) ROS and MDA levels in brain tissue of mice infected with the RH strain of *T. gondii*. (**C**,**D**) ROS and MDA levels in liver tissue of mice infected with the RH strain of *T. gondii*. (**E**,**F**) ROS and MDA levels in brain tissue of mice infected with the PRU strain of *T. gondii*. (**G**,**H**) ROS and MDA levels in liver tissue of mice infected with the PRU strain of *T. gondii*. Data are presented as mean ± SD (*n* = 3). An asterisk denotes a statistically significant difference between groups (* *p* < 0.05, ** *p* < 0.01, *** *p* < 0.001).

The levels of ROS and MDA in the brain and liver tissues of mice infected with RH and PRU strains of *T. gondii* were quantified using ELISA. The results demonstrated that the infection with RH and PRU strains of *T. gondii* led to elevated levels of ROS and MDA in both the brain and liver tissues of mice. The level of ROS in the brain tissue of mice infected with the RH strain of *T. gondii* was significantly elevated at 96 h post-infection and for MDA at 72 h (Figure 1A,B). In liver tissue, ROS levels significantly increased at 96 h, while MDA levels showed significant increases at 6, 24, 72, and 96 h (Figure 1C,D). Additionally, in mice infected with the PRU strain, ROS and MDA levels in brain tissue were significantly different at 14 and 21 days post-infection (Figure 1E,F). ROS levels in liver tissue were significantly elevated at 14 and 21 days, and MDA levels were significantly increased at 7, 14, and 21 days (Figure 1G,H). Our findings indicate that ROS and MDA levels in the brain and liver tissues of mice were significantly elevated at certain time points following *T. gondii* infection, suggesting that this infection may induce ferroptosis in mouse cells.

### 3.2. T. gondii Infection Modulates the Expression of Antioxidant System-Related Genes in Murine Tissues

**Figure 2:**
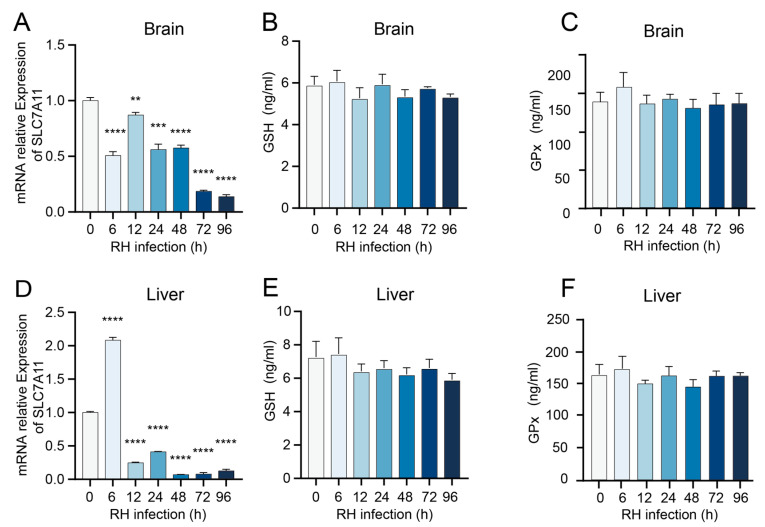
Expression of genes associated with the antioxidant system in brain and liver tissues of mice infected with the *T. gondii* RH strain. (**A**–**C**) Expression levels of SLC7A11, GSH, and GPx in mouse brain tissue. (**D**–**F**) Expression levels of SLC7A11, GSH, and GPx in mouse liver tissue. Data are presented as mean ± SD (*n* = 3). An asterisk denotes a statistically significant difference between groups (** *p* < 0.01, *** *p* < 0.001, **** *p* < 0.0001).

Given the pivotal role of Solute Carrier Family 7 Member 11 (SLC7A11) in regulating cystine uptake for glutathione biosynthesis and ferroptosis resistance, the mRNA transcription levels of SLC7A11 in the brain and liver tissues of mice infected with the *T. gondii* RH strain were quantified using RT-qPCR. Additionally, the expression levels of GSH and GPx were measured via ELISA. The results demonstrated that the mRNA levels of SLC7A11 in both brain and liver tissues were significantly reduced after 12 h of infection (Figure 2A,D). However, no significant alterations were observed in the expression levels of GSH and GPx in either brain or liver tissues (Figure 2B,C,E,F).

**Figure 3:**
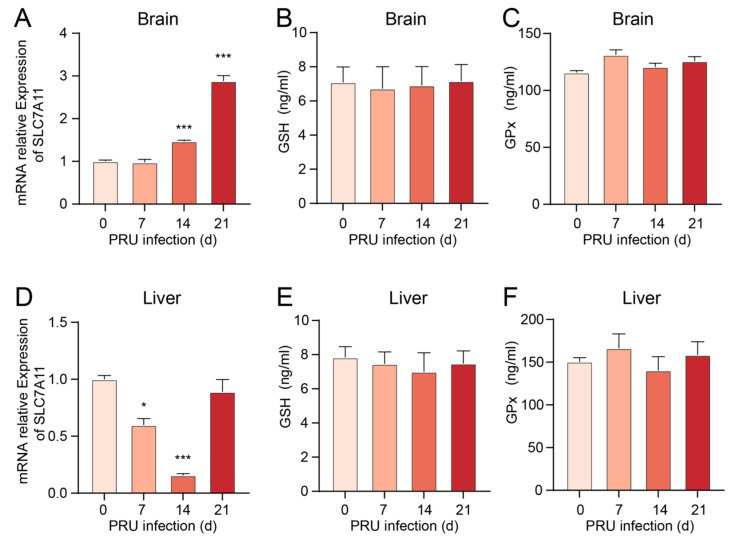
Expression of genes associated with the antioxidant system in brain and liver tissues of mice infected with the *T. gondii* PRU strain. (**A**–**C**) Expression levels of SLC7A11, GSH, and GPx in mouse brain tissue. (**D**–**F**) Expression levels of SLC7A11, GSH, and GPx in mouse liver tissue. Data are presented as mean ± SD (*n* = 3). An asterisk denotes a statistically significant difference between groups (* *p* < 0.05, *** *p* < 0.001).

Furthermore, the expression levels of SLC7A11, GSH, and GPx in both brain and liver tissues of mice infected with the *T. gondii* PRU strain were also assessed. The results demonstrated that the expression level of SLC7A11 in mouse brain tissue exhibited a significant increase at both 14 and 21 days (Figure 3A), whereas no notable alterations were observed in the levels of GSH and GPx (Figure 3B,C). In contrast, the expression level of SLC7A11 in liver tissue showed a marked decrease at 7 and 14 days (Figure 3D), while GSH and GPx levels remained unchanged (Figure 3E,F).

### 3.3. T. gondii Infection Reduces the Expression of GPX4, a Key Negative Regulator of Ferroptosis

**Figure 4:**
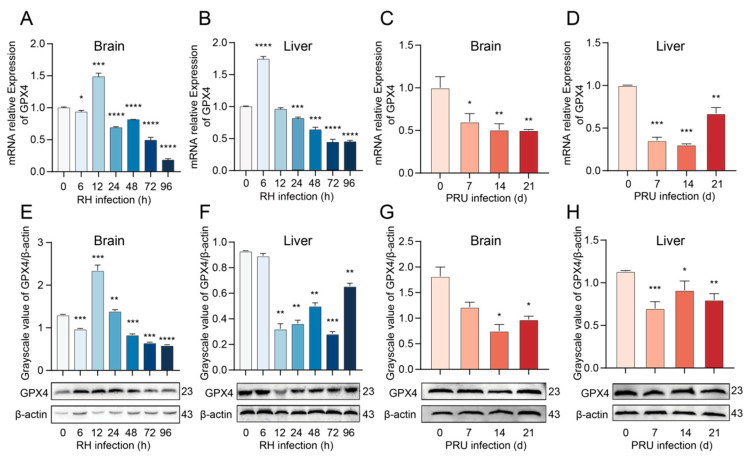
The mRNA and protein expression levels of GPX4 in brain and liver tissues of mice infected with *T. gondii* were investigated. (**A**,**B**) GPX4 mRNA expression levels in brain and liver tissues of mice infected with the RH strain. (**C**,**D**) GPX4 mRNA expression levels expression levels in brain and liver tissues of mice infected with the PRU strain. (**E**,**F**) GPX4 protein expression levels in brain and liver tissues of mice infected with the RH strain. (**G**,**H**) GPX4 protein expression levels in brain and liver tissues of mice infected with the PRU strain. β-actin was utilized as the internal control for quantifying protein expression levels. The WB bands were quantified using ImageJ 1.53q software. Data are presented as mean ± SD (*n* = 3). An asterisk denotes a statistically significant difference between groups (* *p* < 0.05, ** *p* < 0.01, *** *p* < 0.001, **** *p* < 0.0001).

We evaluated the mRNA and protein levels of GPX4, a negative regulator of ferroptosis, in various tissues of mice infected with the RH and PRU strains of *T. gondii*. RT-qPCR analysis revealed that, except at 12 h post-infection, the mRNA expression of GPX4 in brain tissue from RH strain-infected mice was significantly reduced (Figure 4A). Additionally, except at 6 h and 12 h post-infection, the mRNA expression of GPX4 in brain tissue from RH strain-infected mice was significantly reduced (Figure 4B). Notably, the mRNA expression of GPX4 in both brain and liver tissues of PRU strain-infected mice was significantly decreased at 7, 14, and 21 days post-infection (Figure 4C,D).

Simultaneously, we validated our findings through WB analysis, and the results were consistent with the mRNA expression levels (Figure 4E–H). These data suggest a potential involvement of GPX4-mediated ferroptosis in *T. gondii*-induced mortality.

### 3.4. Alterations in mRNA Transcription Levels of ACSL4 and TFR1 Genes in Mice Infected with T. gondii

**Figure 5:**
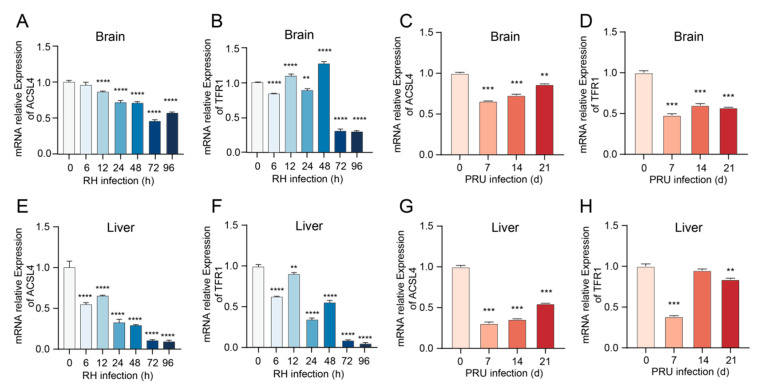
The mRNA expression levels of ACSL4 and TFR1 in brain and liver tissues of mice infected with *T. gondii* were investigated. (**A**,**B**) Expression levels of ACSL4 and TFR1 in the brain tissue of mice infected with the *T. gondii* RH strain. (**C**,**D**) Expression levels of ACSL4 and TFR1 in the brain tissue of mice infected with the *T. gondii* PRU strain. (**E**,**F**) Expression levels of ACSL4 and TFR1 in the liver tissue of mice infected with the *T. gondii* RH strain. (**G**,**H**) Expression levels of ACSL4 and TFR1 in the liver tissue of mice infected with the *T. gondii* PRU strain. Data are presented as mean ± SD (*n* = 3). An asterisk denotes a statistically significant difference between groups (** *p* < 0.01, *** *p* < 0.001, **** *p* < 0.0001).

As key regulators of ferroptosis, Acyl-CoA Synthetase Long-Chain Family Member 4 (ACSL4) and Transferrin Receptor 1 (TFR1) contribute to lipid peroxidation and iron uptake, respectively. Therefore, the mRNA expression levels of ACSL4 and TFR1 in the brain and liver tissues of *T. gondii*-infected mice were assessed using RT-qPCR. The results indicated that the mRNA transcription levels of ACSL4 in the brain tissue of RH-infected mice were significantly reduced after 12 h post-infection (Figure 5A). The mRNA transcription levels of TFR1 showed a significant increase at 12 and 48 h but decreased significantly at other time points (Figure 5B). In liver tissue, the mRNA transcription levels of both ACSL4 and TFR1 were significantly reduced (Figure 5E,F). Following infection with the PRU strain, the mRNA transcription levels of ACSL4 in both liver and brain tissues were significantly decreased (Figure 5C,G). Additionally, the mRNA transcription levels of TFR1 in brain tissue were significantly reduced (Figure 5D), while in liver tissue, TFR1 mRNA levels were significantly decreased at all time points except for day 14, where no significant difference was observed (Figure 5H). The decreased expression levels of ACSL4 and TFR1 during iron-dependent lipid peroxidation did not result in elevated levels of ROS and MDA. Collectively, our findings demonstrate that *T. gondii*-induced ferroptosis in host cells operates independently of ACSL4 and TFR1 modulation, suggesting alternative pathways dominate this regulated cell death process.

### 3.5. T. gondii Infection Triggers Ferroptosis in Host Cells, Thereby Facilitating Its Proliferation Within the Host Organism

**Figure 6:**
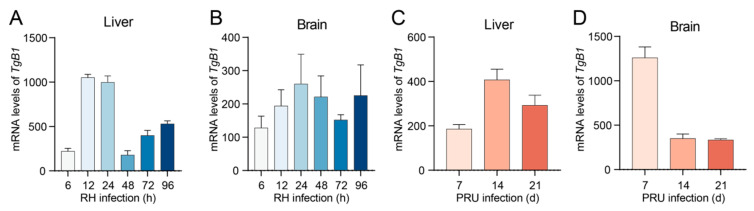
Parasite load in liver and brain of mice. (**A**) Parasite load in the liver tissue of mice infected with the *T. gondii* RH strain. (**B**) Parasite load in the brain tissue of mice infected with the *T. gondii* RH strain. (**C**) Parasite load in the liver tissue of mice infected with the *T. gondii* PRU strain. (**D**) Parasite load in the brain tissue of mice infected with the *T. gondii* PRU strain. Data are presented as mean ± SD (*n* = 3).

To verify the proliferation of *T. gondii* within the host, we conducted RT-qPCR assays to measure the expression levels of the B1 gene in *T. gondii*. The results demonstrated that following the infection of mice with the RH strain, the parasite load was detectable in both brain and liver tissues, exhibiting a general upward trend (Figure 6A,B). Similarly, an upward trend was observed in liver tissues infected with the PRU strain (Figure 6C). These findings suggest that *T. gondii* infection may promote its proliferation by inducing ferroptosis in host cells. Notably, in the brain, B1 gene expression exhibited a sharp increase on day 7 post-infection, followed by stabilization on days 14 and 21 (Figure 6D). We hypothesize that the host’s immune system is likely to become progressively activated as the infection advances. Alternatively, in later stages of infection, *T. gondii* may slow its proliferation rate to adapt to alterations in the host environment.

### 3.6. T. gondii Infection Suppresses the Expression of GPX4 in Vero Cells, Leading to an Elevated Parasite Load

**Figure 7:**
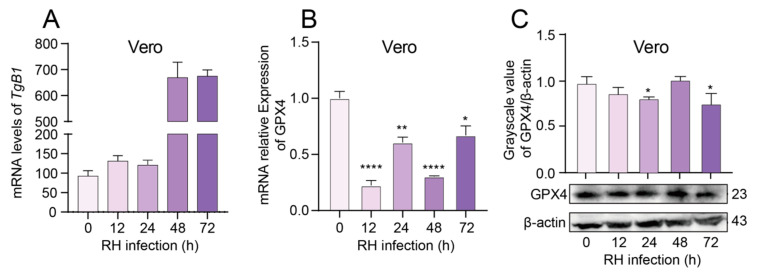
Alterations in GPX4 expression in Vero cells infected with *T. gondii*. (**A**) Parasite load in Vero cells infected with the *T. gondii* RH strain. (**B**) Expression levels of GPX4 mRNA in Vero cells following infection with *T. gondii*. (**C**) Expression levels of GPX4 protein in Vero cells following infection with *T. gondii*. β-actin was utilized as the internal control for quantifying protein expression levels. The WB bands were quantified using ImageJ 1.53q software. An asterisk denotes a statistically significant difference between groups (* *p* < 0.05, ** *p* < 0.01, **** *p* < 0.0001).

The *T. gondii* B1 gene copy number was quantified by RT-qPCR to assess parasite proliferation in Vero cells. The results demonstrated that the parasite load was detectable in Vero cells following infection, with the number of *T. gondii* exhibiting a progressively increasing trend as the duration of infection extended (Figure 7A). The expression levels of GPX4 in Vero cells infected with *T. gondii* were assessed using RT-qPCR and WB analysis. The results indicated a significant reduction in the mRNA expression level of GPX4 following infection with *T. gondii* (Figure 7B). WB data revealed that the protein expression of GPX4 was significantly decreased at 24 and 72 h post-infection (Figure 7C). The inverse correlation between GPX4 downregulation and parasite proliferation suggests a potential role of GPX4-mediated pathways in restricting *T. gondii* replication.

### 3.7. Construction and Validation of Recombinant pVAX-GPX4 Plasmid Expression in Vero Cells

**Figure 8:**
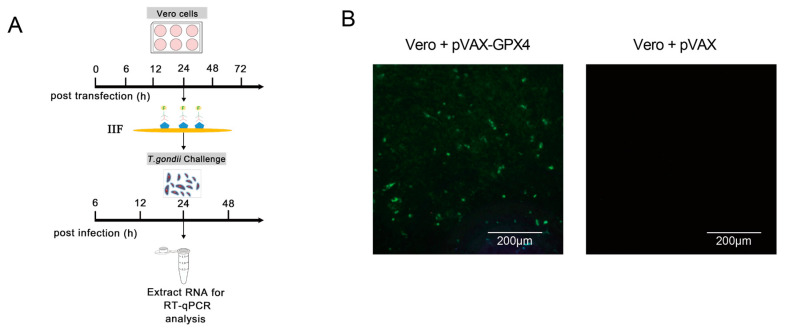
Validation of recombinant pVAX-GPX4 plasmid expression in Vero cells. (**A**) Flowchart for experimental procedures. (**B**) Cells transfected with the pVAX-GPX4 plasmid were compared to those transfected with the pVAX1 empty vector. The scale bar represents 200 μm.

We successfully constructed the recombinant pVAX-GPX4 plasmid utilizing plasmid double-enzyme digestion, and subsequent experiments were conducted in accordance with the schematic diagram presented in Figure 8A. Specifically, the pVAX-GPX4 recombinant plasmid was transfected into Vero cells to assess expression levels, with the pVAX1 empty plasmid serving as a negative control. IIF assays revealed that Vero cells transfected with the pVAX-GPX4 plasmid exhibited distinct green fluorescence, whereas those transfected with the pVAX1 empty plasmid did not display significant green fluorescence (Figure 8B). These results confirm that GPX4 can be effectively expressed in eukaryotic cells.

### 3.8. The Inhibitory Effect of GPX4 Overexpression on the Proliferation of T. gondii RH Strain in Vero Cells

**Figure 9:**
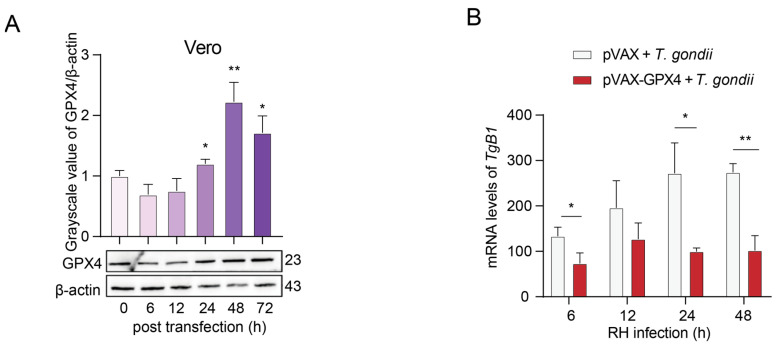
Impact of GPX4 overexpression on the proliferation of *T. gondii*. (**A**) Expression of GPX4 protein in Vero cells transfected with the pVAX-GPX4 plasmid at various time points. β-actin was utilized as the internal control for quantifying protein expression levels. The WB bands were quantified using ImageJ 1.53q software. (**B**) Results of the RT-qPCR method for the detection of *T. gondii* levels in Vero cells. An asterisk denotes a statistically significant difference between groups (* *p* < 0.05, ** *p* < 0.01).

The protein level of GPX4 in Vero cells transfected with the pVAX-GPX4 plasmid was evaluated using WB analysis to determine the optimal time point for transfection. The results indicated that the GPX4 protein levels in Vero cells transfected with the pVAX-GPX4 plasmid at 24, 48, and 72 h post-transfection were significantly elevated, leading to the selection of 24 h as the optimal transfection time (Figure 9A). Subsequently, cells were infected with the *T. gondii* RH strain 24 h after transfection with the plasmid, and the parasite load was quantified using RT-qPCR. The findings revealed that the expression level of the *T. gondii* B1 gene in Vero cells overexpressing GPX4 was significantly lower compared to control cells at 6, 24, and 48 h post-infection (Figure 9B). The results demonstrated that the overexpression of GPX4 significantly suppressed the proliferation of *T. gondii* within Vero cells.

## 4. Discussion

Iron is a critical nutrient essential for the survival of *T. gondii*; however, an excess of iron can result in iron toxicity [31,32,33,34]. During the process of parasite infection, the host employs nutritional immunity strategies, metal restriction, and toxicity, to inhibit the invasion and proliferation of the parasite. Meanwhile, the parasite can survive in environments characterized by metal depletion or excess through adaptive mechanisms [35]. Our study bridges this interplay with ferroptosis driven by iron-dependent lipid peroxidation.

As a novel form of cell death [36], the discovery of ferroptosis has ushered in a new horizon for the investigation of parasitic diseases, and its clinical significance is increasingly evident in the pathogenesis, progression, and therapeutic approaches for these conditions. Toxoplasmosis poses a significant threat to global public health security [37]. Our research points towards an in-depth investigation into the relationship between ferroptosis and *T. gondii*, along with the targeted modulation of cellular ferroptosis, which may emerge as a promising strategy for the control and prevention of toxoplasmosis.

In this study, we conducted an in-depth investigation into the series of changes in mice infected with *T. gondii* RH and PRU strains as well as in Vero cells. Significant findings were uncovered regarding the mechanisms of *T. gondii* infection and its relationship with host cell ferroptosis and the associated regulatory pathways. The results demonstrated that the infection with RH and PRU strains of *T. gondii* led to a significant increase in the expression levels of ROS and MDA in both brain and liver tissues of the host mice. This strongly indicates that *T. gondii* infection induces ferroptosis in host cells. Ferroptosis represents a novel form of iron-dependent programmed cell death that distinctly differs from apoptosis, necrosis, and autophagy. A hallmark feature of ferroptosis is the accumulation of intracellular ROS and lipid peroxidation products, such as MDA [10,17]. These findings are in agreement with prior research, where Huang et al. [38] demonstrated that the combination of dihydroartemisinin and the ferroptosis inducer, RSL3, significantly enhanced ROS accumulation and effectively inhibited the growth of *T. gondii*. Our study establishes a connection between *T. gondii* infection and ferroptosis in host cells, thereby enhancing our understanding of the pathogenic mechanisms of *T. gondii*. Currently, research on ferroptosis predominantly centers on its functional implications in diseases such as cancer and metabolic disorders [39], with limited exploration into the effects of parasitic infections. Notably, it remains inconclusive whether *T. gondii* infection can induce ferroptosis in host cells, and there is a lack of international reports addressing this specific issue. The majority of studies on the mechanisms of *T. gondii* infection have primarily concentrated on immune evasion and inflammatory responses [40]. However, our findings uncover a novel mechanism at the level of cell death regulation, offering a fresh perspective for future research endeavors.

Further studies demonstrated that the mRNA and protein expression levels of GPX4, a key regulator of ferroptosis, were significantly downregulated in the brain and liver tissues of mice following infection, while the parasitic load of *T. gondii* was concurrently increased. This indicates that *T. gondii* infection suppresses GPX4 expression, thereby impairing its ability to clear lipid peroxides, ultimately facilitating the proliferation of *T. gondii*. GPX4, a unique selenoprotein, has been identified as a critical enzyme in the reduction of phospholipid hydroperoxides (PLOOH) in mammalian cells [41,42]. The enzymatic activity of GPX4 is critically dependent on its catalytic selenocysteine residues, which neutralize PLOOH using electrons supplied by GSH. Subsequently, oxidized glutathione (GSSG) is recycled to its reduced form via the provision of electrons from NADPH by glutathione–disulfide reductase (GSR). When any of these processes are disrupted, such as when Erastin inhibits GSH synthesis by blocking SLC7A11 activity, which in turn affects intracellular cystine transport, a decrease in GSH levels leads to inhibition of GPX4 activity. Consequently, the cell loses its capacity to counteract PLOOH accumulation, ultimately resulting in ferroptosis [22]. As a pivotal protein in the regulation of ferroptosis, GPX4 maintains intracellular redox homeostasis and inhibits ferroptosis through the catalysis of glutathione to reduce lipid peroxides [43]. Recent studies have provided further evidence that mice infected with acute lymphocytic choriomeningitis virus (ALCMV) exhibit inhibited GPX4 expression in T cells. It was observed that ALCMV-infected mice with suppressed GPX4 expression exhibited significantly higher mortality compared to ALCMV-infected mice with normal GPX4 expression. Additionally, the administration of autophagy inhibitors, necrosis inhibitors, and apoptosis inhibitors did not reverse this increased mortality. However, the addition of Fer-1 and DFO could mitigate the death of GPX4-deficient T cells caused by viral infection. Following the inhibition of GPX4 expression, membrane lipid peroxides accumulated continuously in T cells post-infection, leading to a decrease in cell viability [44]. These findings suggest that GPX4 plays a protective role in T cells during ALCMV infection [44]. YANG et al. [21] demonstrated that cells exhibiting reduced GPX4 expression exhibited increased sensitivity to ferroptosis, whereas cells with elevated GPX4 expression displayed enhanced resistance to this form of cell death. Our results indicate that *T. gondii* may disrupt the redox balance of host cells by impairing the normal function of GPX4, thereby creating an environment favorable for its proliferation. This discovery elucidates the molecular mechanism underlying *T. gondii*-induced ferroptosis in host cells and offers valuable insights into the survival and reproductive strategies of *T. gondii* within the host.

Furthermore, the mRNA and protein expression levels of GPX4 in Vero cells were significantly diminished following infection with the RH strain of *T. gondii*. This observation corroborates that the suppressive effect of *T. gondii* infection on GPX4 expression is consistent across different cellular models, thereby enhancing the robustness and generalizability of the study findings. Moreover, when *T. gondii* RH strains were exposed to GPX4-overexpressing Vero cells, the parasite burden was significantly reduced, which directly indicates that GPX4 overexpression can inhibit *T. gondii* proliferation. These findings provide retrospective validation of GPX4′s critical regulatory role in *T. gondii* infection and proliferation.

**Figure 10:**
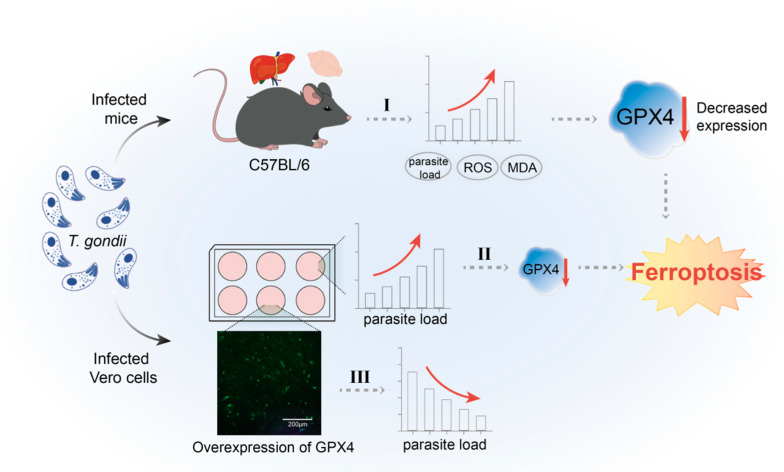
Schematic representation of the mechanism by which *T. gondii* infection modulates host cell ferroptosis via GPX4 regulation. (I) The levels of ROS and MDA in the liver and brain increased significantly after *T. gondii* infection, GPX4 levels decreased notably, and an increase in the parasitic load was concurrently observed. (II) After *T. gondii* infection with Vero cells, the level of GPX4 was notably decreased, and an increase in the parasite load was concurrently observed. (III) Overexpression of GPX4 in Vero cells significantly decreased the parasitic load of *T. gondii*.

Despite the significant progress made in this study, several limitations should be acknowledged. Firstly, the investigation was confined to mouse models and Vero cell lines. Future research should extend to additional animal models and a broader range of cell types to comprehensively assess the relationship between *T. gondii* infection, ferroptosis, and GPX4 regulation. Secondly, while it is established that *T. gondii* infection enhances self-proliferation and induces ferroptosis by inhibiting GPX4 expression (Figure 10), the precise molecular mechanisms underlying this inhibition remain elusive. Specifically, it is unclear whether virulence factors or signaling pathways are involved in regulating GPX4 expression, which warrants further investigation.

## 5. Conclusions

In summary, this study uncovered the phenomenon of ferroptosis induced by *T. gondii* infection in host cells and elucidated the critical regulatory role of GPX4 in this process, as well as its relationship with *T. gondii* proliferation. These findings provide a novel theoretical foundation for further understanding the pathogenic mechanisms of *T. gondii* and offer potential regulatory factors and insights for developing innovative prevention and control strategies against *T. gondii* infection.

## Figures and Tables

**Table 1:** The primers used in this study.

Primers	Sequences (5′–3′)
GPX4-F	AAAC↓TGCAGATGAACCTCGGCCGCCTTTG
GPX4-R	CTAGT↓CTAGACTAGAAATAGTGGGGCAGGTCCT
GPX4-qPCR-F	ATAAGAACGGCTGCGTGGTGAAG
GPX4-qPCR-R	TAGAGATAGCACGGCAGGTCCTTC
SLC7A11-qPCR-F	CATGGTTGTCCTCTCCCTTTAC
SLC7A11-qPCR-R	ACTTGGGTTTCTTGTCCCATAC
ACSL4-qPCR-F	TATGGGCTGACAGAATCATG
ACSL4-qPCR-R	CAACTCTTCCAGTAGTGTAG
TFR1-qPCR-F	TGAACCTGGACTATGAGATG
TFR1-qPCR-R	TAGAAGTAGCACGGAAGTAG
B1-qPCR-F	ACGACATCGCATTCAAGGGA
B1-qPCR-R	CATGAGAGGAGGCAGCACAA
β-actin-qPCR-F	GGCTGTATTCCCCTCCATCG
β-actin-qPCR-R	CCAGTTGGTAACAATGCCATGT

Note: The underscore denotes the cleavage site, while the arrow signifies the direction of action of the restriction enzyme.

## Data Availability

Available upon request to interested researchers.
